# Novel insight into atogepant mechanisms of action in migraine prevention

**DOI:** 10.1093/brain/awae062

**Published:** 2024-02-27

**Authors:** Agustin Melo-Carrillo, Andrew M Strassman, Ron Broide, Aubrey Adams, Brett Dabruzzo, Mitchell Brin, Rami Burstein

**Affiliations:** Department of Anesthesia, Critical Care and Pain Medicine, Beth Israel Deaconess Medical Center. Boston, MA 02115, USA; Harvard Medical School, Harvard University, Boston, MA 02115, USA; Department of Anesthesia, Critical Care and Pain Medicine, Beth Israel Deaconess Medical Center. Boston, MA 02115, USA; Harvard Medical School, Harvard University, Boston, MA 02115, USA; Allergan, an Abbvie Company, Irvine, CA 92612, USA; Allergan, an Abbvie Company, Irvine, CA 92612, USA; Allergan, an Abbvie Company, Irvine, CA 92612, USA; Allergan, an Abbvie Company, Irvine, CA 92612, USA; Department of Neurology, University of California, Irvine, CA 92697USA; Department of Anesthesia, Critical Care and Pain Medicine, Beth Israel Deaconess Medical Center. Boston, MA 02115, USA; Harvard Medical School, Harvard University, Boston, MA 02115, USA

**Keywords:** migraine, headache, trigeminovascular, gepants, central sensitization, pain

## Abstract

Recently, we showed that while atogepant—a small-molecule calcitonin gene-related peptide (CGRP) receptor antagonist—does not fully prevent activation of meningeal nociceptors, it significantly reduces a cortical spreading depression (CSD)-induced early response probability in C fibres and late response probability in Aδ fibres. The current study investigates atogepant effect on CSD-induced activation and sensitization of high threshold (HT) and wide dynamic range (WDR) central dura-sensitive trigeminovascular neurons.

In anaesthetized male rats, single-unit recordings were used to assess effects of atogepant (5 mg/kg) versus vehicle on CSD-induced activation and sensitization of HT and WDR trigeminovascular neurons.

Single cell analysis of atogepant pretreatment effects on CSD-induced activation and sensitization of central trigeminovascular neurons in the spinal trigeminal nucleus revealed the ability of this small molecule CGRP receptor antagonist to prevent activation and sensitization of nearly all HT neurons (8/10 versus 1/10 activated neurons in the control versus treated groups, *P =* 0.005). In contrast, atogepant pretreatment effects on CSD-induced activation and sensitization of WDR neurons revealed an overall inability to prevent their activation (7/10 versus 5/10 activated neurons in the control versus treated groups, *P =* 0.64). Unexpectedly however, in spite of atogepant’s inability to prevent activation of WDR neurons, it prevented their sensitization (as reflected their responses to mechanical stimulation of the facial receptive field before and after the CSD).

Atogepant’ ability to prevent activation and sensitization of HT neurons is attributed to its preferential inhibitory effects on thinly myelinated Aδ fibres. Atogepant’s inability to prevent activation of WDR neurons is attributed to its lesser inhibitory effects on the unmyelinated C fibres. Molecular and physiological processes that govern neuronal activation versus sensitization can explain how reduction in CGRP-mediated slow but not glutamate-mediated fast synaptic transmission between central branches of meningeal nociceptors and nociceptive neurons in the spinal trigeminal nucleus can prevent their sensitization but not activation.

## Teaser

Melo-Carrillo *et al.* report that atogepant prevents sensitization of all classes of central trigeminovascular neurons, but not their activation. This finding makes it possible to distinguish between drugs that prevent migraine onset and those that prevent disease progression—as in the absence of sensitization the disease is far less likely to progress.

## Introduction

Calcitonin gene-related peptide (CGRP) is thought to play a critical role in the pathogenesis of migraine headache.^1^ The notion that CGRP is involved in the generation of the headache phase of migraine is based on its release from nerve endings of sensory trigeminal ganglion neurons in the dura,^2,3^ pia^4^ and the superficial layers of the spinal trigeminal nucleus (STN),^5^ its ability to trigger a migraine-like headache in patients,^6^ its ability to dilate the middle meningeal artery^7^ and the high efficacy of drugs that inhibit peripheral CGRP signalling [e.g. anti-CGRP-mAbs (monoclonal antibodies), anti-CGRP/R-mAbs, CGRP receptor antagonists] in migraine prevention^8-12^ and resolution.^13,14^ In agreement with these clinical studies, behavioural studies in rodents have found therapeutic effects of CGRP inhibitors in several models of headache, including systemic glyceryl trinitrate (GTN),^15-18^ dural application of inflammatory mediators^19^ or potassium chloride,^20,21^ medication overuse headache,^22^ CGRP-induced photophobia^23^ and umbellulone-induced hyperalgesic priming.^24^ Similarly, physiological studies found inhibitory effects of these agents in response to systemic administration of GTN or other nitric oxide donors,^25-27^ direct electrical or chemical stimulation of the dura^28,29^ and cortical spreading depression (CSD).^30-32^

Relevant to the current study, we showed recently that pretreatment with atogepant—a small molecule CGRP receptor antagonist approved recently for migraine prevention—produced delayed and relatively prolonged (90–210 min after occurrence of CSD) inhibition of neuronal firing in thinly myelinated meningeal Aδ fibres and immediate but relatively brief (0–30 min after occurrence of CSD) inhibition of firing in the unmyelinated meningeal C fibres.^30^ These data follow a recent line of publications showing that CSD activates C fibres earlier than it activates Aδ fibres,^33^ that fremanezumab—an anti-CGRP monoclonal antibody (anti-CGRP-mAb)—prevents activation of meningeal Aδ but not C fibres and high threshold (HT) but not wide dynamic range (WDR) dura-sensitive trigeminovascular neurons in STN,^31,32^ that peptidergic (presumably C-fibre) neurons in the trigeminal ganglion contain CGRP^34-37^ but not the CGRP receptor, and that non-peptidergic (mostly Aδ-fibre) neurons express the CGRP receptor.^34,36,37^

Given atogepant's inhibitory effects on the two populations of meningeal nociceptors, the goal of the current study was to investigate the effects of atogepant on activation and sensitization of the two populations of nociceptive (WDR and HT) trigeminovascular neurons in the STN. As in other studies using this model,^30-32^ neuronal activation was evoked by CSD, a cortical event that has been implicated in the initiation of migraine attacks^38^ and the activation of peripheral and central trigeminovascular neurons.^33,39^

## Materials and methods

Experiments were approved by the Beth Israel Deaconess Medical Center and Harvard Medical School standing committees on animal care and were in accordance with the US National Institutes of Health's ‘Guide for the Care and Use of Laboratory Animals’. A total of 40 male Sprague-Dawley rats (240–300 g) were used.

### Overview of experimental protocol

**Figure 1:**
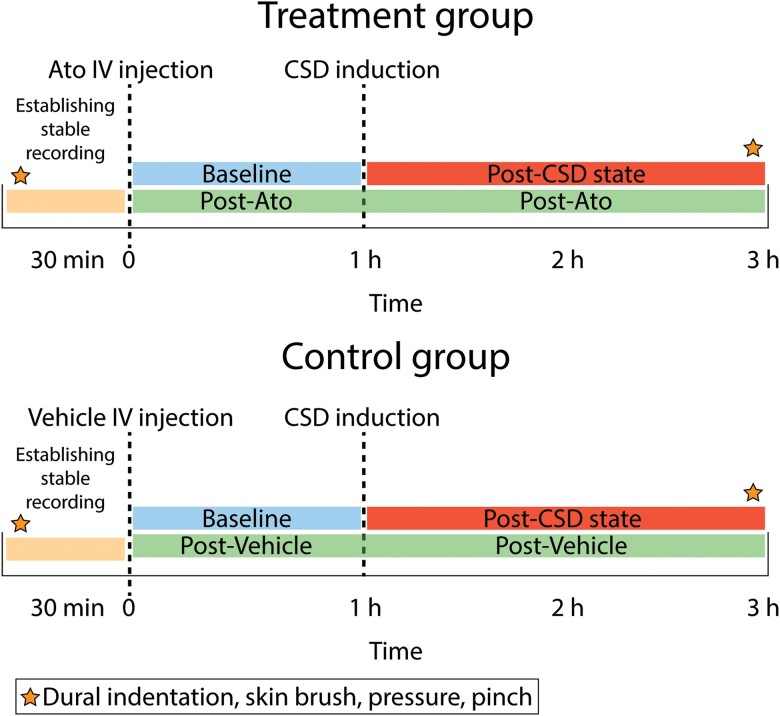
**Experimental design.** Treatment group received intravenous atogepant (Ato). Control group received intravenous (IV) saline (vehicle). Stars depict times at which responses to mechanical stimulation of the dura and skin were determined. CSD = cortical spreading depression.

An experimental protocol was designed to test the effect of atogepant on the activity of central trigeminovascular neurons in the dorsal horn (spontaneous and induced activity in response to peripheral stimulation and CSD) (Fig. 1). Based on the time course of action, atogepant was administered during the neuronal recording session, 1 h prior to the induction of CSD. Two groups of rats were studied with this protocol: (i) atogepant; and (ii) vehicle. In this protocol, single-unit recordings of activity of central trigeminovascular neurons were obtained in anaesthetized rats. Thirty minutes after characterization of responses to dural and facial stimulation, rats received an intravenous (IV) infusion of atogepant or vehicle and their spontaneous activity was recorded for 1 h. CSD was then induced and recording of neuronal activity continued for two more hours. Characterization of responses to dural and facial stimulation was repeated 2 h after CSD induction (Fig. 1). Ongoing discharge was recorded continuously throughout the experiment. Methodological details are described below.

### Surgical preparation

Rats were anaesthetized with intraperitoneal urethane (0.9–1.2 g/kg) and surgically prepared for recording of neuronal activity in the STN, as described in detail previously.^31^ The rats were intubated to allow artificial ventilation, and the femoral vein was cannulated for drug infusion. Core temperature and end-tidal CO_2_ were monitored and kept within physiological range. Rats were paralyzed with rocuronium and ventilated. Data were used only in cases in which the physiological condition of the rats (heart rate, blood pressure, respiration, end-tidal CO_2_) and the neuronal isolation signal (signal-to-noise ratio ∼1:3) were stable throughout the experimental period. A craniotomy was made to expose the left transverse sinus and a second craniotomy was made in the left parietal bone to allow recording of electrocorticogram activity and induction of CSD by pinprick. A segment of the spinal cord between the obex and C2 was exposed for recording of activity from central neurons in the left STN (C1–2 dorsal horn).

### Identification and characterization of central trigeminovascular neurons

To record neuronal activity, a tungsten microelectrode (impedance 1–4 MΩ, FHC Co.) was lowered repeatedly into the STN (C1–2 dorsal horn) in search of central trigeminovascular neurons receiving input from the dura. Trigeminovascular neurons were first identified based on their responses to electrical stimulation of the dura. They were selected for the study if they exhibited discrete firing bouts in response to ipsilateral electrical (0.1–3.0 mA, 0.5 ms, 0.5 Hz pulses) and mechanical (with calibrated von Frey monofilaments) stimulation of the exposed cranial dura.

Dural receptive fields were mapped by indenting the dura [4.19 g von Frey hair (VF) monofilament]. Cutaneous receptive fields were mapped by applying innocuous and noxious mechanical stimulation to all facial skin areas, as described previously.^31^ Responses to mechanical stimulation of the skin were determined by applying brief (10 s) innocuous and noxious stimuli to the most sensitive portion of the cutaneous receptive field. Innocuous stimuli consisted of slowly passing a soft bristled brush across the cutaneous receptive field and pressure applied with a loose arterial clip. Noxious stimuli consisted of pinch with a strong arterial clip.^31^ Two classes of neurons were thus identified: WDR neurons (incrementally responsive to brush, pressure and pinch) and HT neurons (unresponsive to brush). A real-time waveform discriminator was used to create and store a template for the action potential evoked in the neuron under study by electrical pulses on the dura; spikes of activity matching the template waveform were acquired and analysed online and offline using Spike 2 software (Cambridge Electronic Design Limited).

At the conclusion of all experiments, a small electrolytic lesion was made at the recording site, and its localization in the dorsal horn was determined post-mortem using histological analysis, as described previously.^31^ Only one neuron was studied in each animal.

### Atogepant infusion

One hour prior to induction of CSD, atogepant or vehicle (50% dextrose water, 40% PEG 400 and 10% tocopherol) were administered intravenously at the same volume and rate. The final dose of atogepant was 5 mg/kg (5 mg/ml, 1 ml/kg, total infusion volume 0.24–0.3 ml, infusion rate 6 ml/min, ∼30 s total). We used our previous published data^40^ to determine how much time should elapse before testing the effects of atogepant on CSD-induced activation.

### CSD induction and electrocorticogram recording

One hour after atogepant infusion, CSD was induced, as previously described.^40^ For verification of CSD, electrocorticogram activity was recorded with a glass micropipette (0.9% saline, ∼1 MΩ, 7 μm tip) placed just below the surface of the parietal cortex (∼100 μm). We opted to use a single wave of CSD because patients rarely describe/experience more than one wave of aura in a single migraine attack; thus, this paradigm adheres better to the clinical reality.^41^

### Data analysis

Analyses of neuronal firing before and after induction of CSD and in responses to mechanical stimulation of the dura and skin, as well as their classification, were carried out by an experimenter who was blinded to the treatment each rat received (i.e. treatment with atogepant or with vehicle). Randomization was applied to recording order of neuronal class (WDR and HT) and treatment (control, atogepant). To determine neuronal responses to CSD, the mean firing frequency occurring before the onset of CSD (calculated from measuring the spontaneous activity during the 1 h period before CSD onset) was compared to the mean firing frequency recorded for one (0–60 min after CSD) and two (recorded from 60–120 min after CSD) hours after CSD induction. A neuron was considered activated if its mean firing rate after CSD exceeded its mean baseline activity by one standard deviation (SD) for a period >10 min, which translated to an ∼>33% increase in activity. To calculate the response to mechanical stimulation of the dura (von Frey hair, VFH) and skin (brush, pressure, pinch), the mean firing frequency occurring before the onset of the first stimulus (mean spontaneous activity for 30 min) was subtracted from the mean firing frequency that occurred throughout the duration of each stimulus. Mean firing rates of respective values were compared using non-parametric repeated measures test (Friedman test) and *post hoc* analysis (Tukey HSD). The level of significance was set at 0.05. To examine the total proportion of responding HT and WDR neurons across the testing conditions, a generalized linear mixed model with a binomial distribution and log link was used to regress response onto treatment (control versus treatment), test (spontaneous activity, VFH, brush, pressure, pinch) and treatment × test interaction. To accommodate repeated measurements, a random intercept was specified at the level of neuron (i.e. each neuron received its own intercept) but dropped if a lack of neuron-level variance was observed.

## Results

### Baseline activity

The median [interquartile range (IQR)] firing rates at baseline, prior to CSD induction, were 9.95 spikes/s (4.47–15.0) for HT neurons in the control group, 6.24 spikes/s (1.8–15.05) for HT neurons in the treatment group, 3.25 spikes/s (0–7.22) for WDR neurons in the control group and 5.75 spikes/s (0.45–10.23) for WDR neurons in the treatment group. As noted below, there was no significant difference in baseline firing between neurons from animals treated with atogepant and neurons from animals treated with vehicle [*P* = 0.62 (*Z* = 0.56, *n*_1_ = 10, *n*_2_ = 10), *P* = 0.84 (*Z* = 0.25, *n*_1_ = 10, *n*_2_ = 10), HT and WDR, respectively, Wilcoxon signed-rank test].

When analysing both types of neurons (HT and WDR) combined, the baseline median (IQR) rates prior to CSD induction was 6.05 spikes/s (1.27–11.7) for all neurons in the control group and 5.75 spikes/s (0.96–11.36) for all neurons in the treatment group. There was no significant difference in baseline firing between neurons from the control group and neurons from the treatment group [*P* = 0.98 (*Z* = 0.03, *n*_1_ = 20, *n*_2_ = 20)], Wilcoxon signed rank test].

### Activation by CSD

#### High threshold neurons

**Figure 2:**
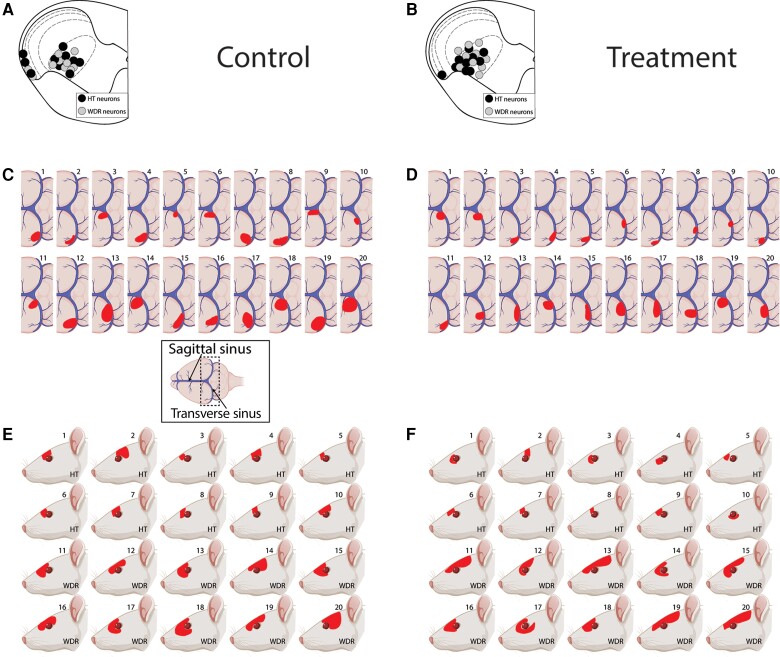
**Recording sites (A and B), dural (C and D) and facial (E and F) receptive fields of all studied trigeminovascular neurons in the spinal trigeminal nucleus**. Black and grey circles depict locations of lesions of high threshold (HT) and wide dynamic range (WDR) neurons in the different laminae of the upper cervical spinal cord segment. Red indicates locations and sizes of most sensitive areas of dural and cutaneous receptive fields. *Inset* in **C** depicts dural receptive field drawings. Created with Biorender.com.

**Figure 3:**
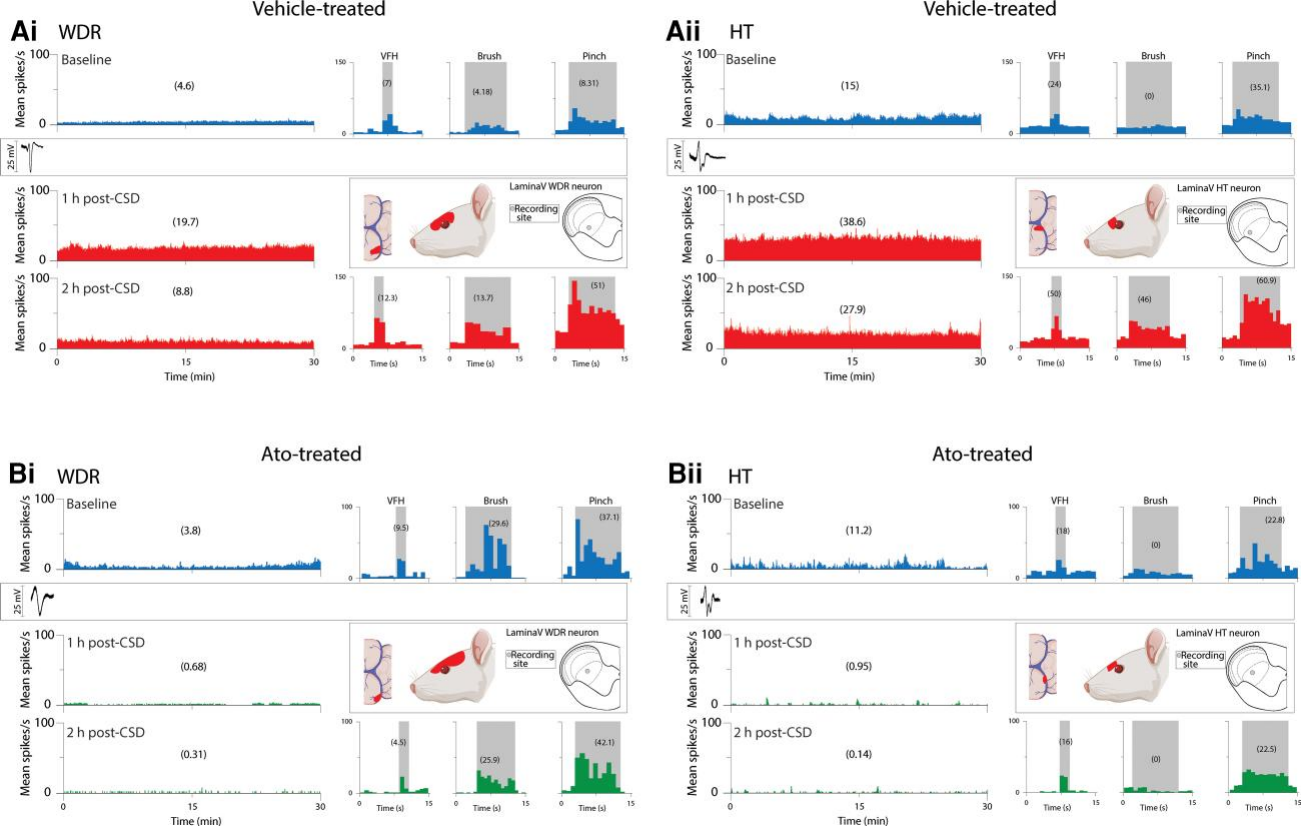
**Examples of CSD effects on activation and sensitization of individual WDR [A(i) and B(i)] and HT [A(ii) and B(ii)] neurons in untreated (vehicle) and treated (atogepant) animals**. [**A**(**i** and **ii**)] Plots of firing rate before (blue) and 1 and 2 h after (red) cortical spreading depression (CSD) induction in an animal treated with saline (vehicle). Note that spontaneous firing and responses to mechanical stimulation of the dura and skin increased after the CSD. [**B**(**i** and **ii**)] Plots of firing rate before (blue) and 1 and 2 h after (green) CSD induction in an animal treated with atogepant. Note that spontaneous activity and responses to stimulation of the dura and skin did not increase after the CSD. Recording sites and locations of dural and cutaneous receptive fields of each of the four neurons are shown. HT = high threshold; VFH = von Frey hair; WDR = wide dynamic range.

**Figure 4:**
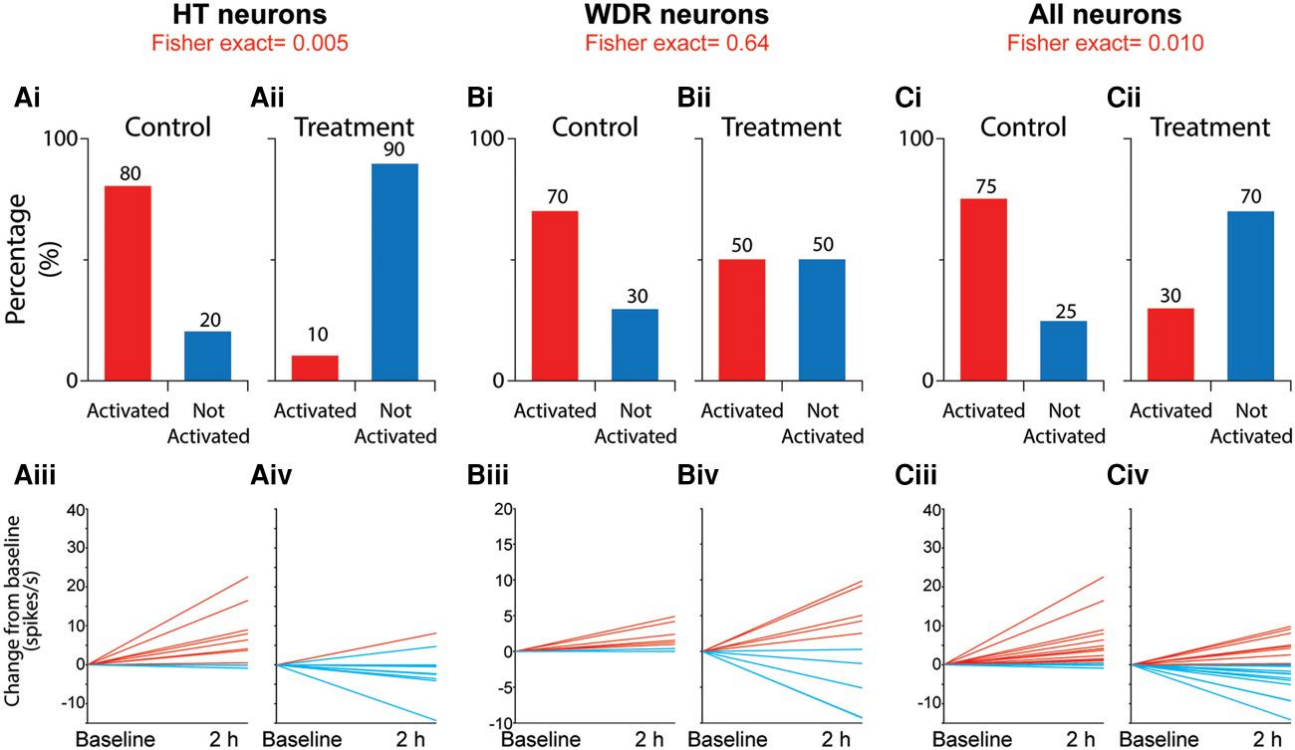
**Percentage of HT, WDR and all neurons activated by CSD in the 10 animals treated with saline [Control, A(i), B(i) and C(i)] and 10 animals treated with atogepant [Treatment, A(ii), B(ii) and C(ii)]**. Fisher exact was used to calculate the level of significance of the percentage differences between the groups. Plots of changes in spontaneous firing rate recorded in individual high threshold (HT) [**A**(**iii** and **iv**)], wide dynamic range (WDR) [**B**(**iii** and **iv**)] and all [**C**(**iii** and **iv**)] neurons before and 2 h after occurrence of cortical spreading depression (CSD). Red and blue lines depict neurons classified as activated and not activated, respectively.

**Figure 5:**
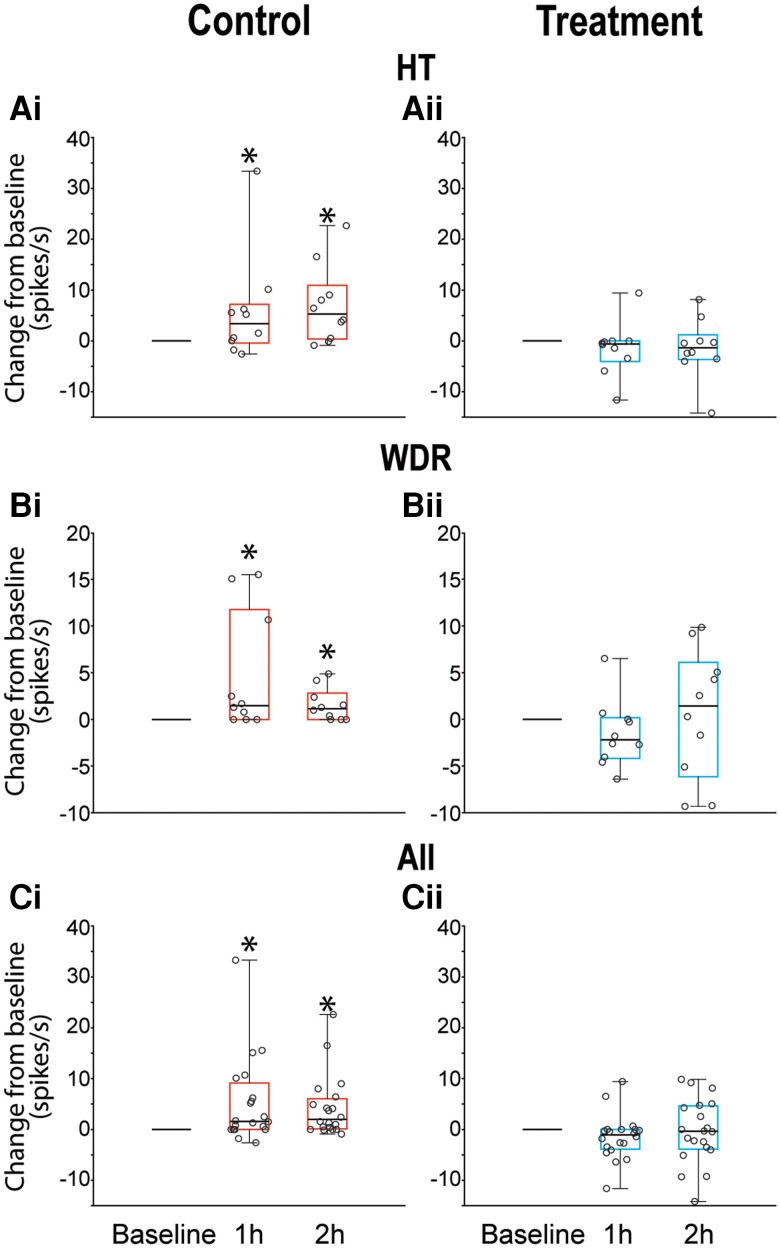
**Spontaneous activity changes (from baseline) recorded in HT [A(i and ii)], WDR [B(i and ii)] and all [C(i and ii)] neurons at 1 and 2 h after CSD induction**. Box and whisker plots depict median and IQR (**A** and **B**, 10 neurons per group; **C**, 20 neurons per group). Scatter plots depict changes from baseline of individual neurons. *P < 0.05 Friedman test; *post hoc*/Tukey HSD. CSD = cortical spreading depression; HT = high threshold; WDR = wide dynamic range.

CSD effects were tested on 20 HT neurons (control group, *n* = 10; treatment group, *n* = 10) in laminae I and V of the STN (Fig. 2A and B). Their receptive fields included the intracranial dura (Fig. 2C and D) and peri-orbital skin (Fig. 2E and F). Typical CSD effects on spontaneous firing and responses to mechanical stimulation of the dura and skin are shown in Fig. 3, where activation and sensitization occur in control [Fig. 3A(ii)] but not treated [Fig. 3B(ii)] animals. In the control group, CSD triggered distinct and prolonged activation in 8/10 (80%) neurons [Fig. 4A(i)], whereas in the treatment group, CSD activated only 1/10 (10%) neurons (*P=* 0.005, χ^2^ = 9.89, DF = 1, Fisher Exact) [Fig. 4A(ii)]. Actual changes in spontaneous firing rate recorded in each HT neuron before and 2 h after occurrence of CSD are shown in Fig. 4A(iii) (control) and Fig. 4A(iv) (treatment). Firing rate analyses of all HT neurons (activated and non-activated) showed that in the control group, baseline spontaneous activity [9.95 spikes/s (4.47–15.0)] [median (IQR)] increased significantly by 3.34 spikes/s (0–7.17) 1 hour after CSD and by 5.25 spikes/s (0.34–10.87) 2 h after CSD (χ^2^ = 7.1, DF = 2, *P =* 0.025, Friedman test). *Post hoc* (Tukey HSD) comparisons between baseline and 1 and 2 h post-CSD onset yielded *P-*values of 0.042 and 0.009, respectively [Fig. 5(Ai)]. In contrast, in the treatment group, spontaneous activity remained unchanged after CSD. The neurons’ baseline firing rate [6.24 spikes/s (1.8–15.05) [median (IQR)] did not change significantly at 1 h [decrease of 0.58 spikes/s (−4.04 to 0) or 2 h (−1.33 spikes/s) (−3.63 to 1.18) after CSD (χ^2^ = 5.15, DF = 2, *P =* 0.96, Friedman test)] [Fig. 5A(ii)].

#### Wide dynamic range neurons

CSD effects were tested on 20 WDR neurons (control group, *n* = 10; treatment group, *n* = 10) in laminae I and V of the STN (Fig. 2A and B). Their receptive fields included the intracranial dura (Fig. 2C and D) and peri-orbital skin (Fig. 2E and F). Typical CSD effects on spontaneous firing and responses to mechanical stimulation of the dura and skin are shown in Fig. 3, where activation and sensitization occur in control [Fig. 3A(i)] but not treated [Fig. 3A(ii)] animals. In the control group, CSD triggered distinct and prolonged activation in 7/10 (70%) neurons [Fig. 4B(i)], whereas in the treatment group, CSD activated 5/10 (50%) neurons (*P =* 0.64, χ^2^ = 0.83, DF = 1, Fisher Exact) [Fig. 4B(ii)]. Actual changes in spontaneous firing rate recorded in each WDR neuron before and 2 h after occurrence of CSD are shown in Fig. 4B(iii) (control) and Fig. 4B(iv) (treatment). Firing rate analyses of all WDR neurons (activated and non-activated) showed that in the control group, baseline spontaneous activity [5.75 spikes/s (0.45–10.23)] increased significantly by 1.5 spikes/s (0–11.8) 1 h after CSD and by 1.15 spikes/s (0–2.85) 2 h after CSD (χ^2^ = 7.8, DF = 2, *P =* 0.0043, Friedman test). *Post hoc* (Tukey HSD) comparisons between baseline and 1 and 2 h post CSD onset yielded *P-*values of 0.023 and 0.026, respectively [Fig. 5B(i)]. In contrast, in the treatment group, spontaneous activity remained unchanged after CSD. The neurons’ baseline firing rate of 3.25 spikes/s (0–7.22) did not change significantly at 1 h [decrease of 2.2 spikes/s (−4.17 to 0.17) or 2 h (1.42 spikes/s) (−6.13 to 6.79) after CSD (χ^2^ = 1.4, DF = 2, *P =* 0.52, Friedman test)] [Fig. 5B(ii)].

#### All neurons

In the control group, CSD triggered distinct and prolonged activation in 15/20 (75%) neurons [Fig. 4C(i)], whereas in the treatment group, CSD activated 6/20 (30%) neurons (*P =* 0.01, χ^2^ = 8.12, DF = 1, Fisher Exact) [Fig. 4C(ii)]. Actual changes in spontaneous firing rate recorded in each HT neuron before and 2 h after occurrence of CSD are shown in Fig. 4C(iii) (control) and Fig. 4C(iv) (treatment). Firing rate analyses of all neurons (activated and non-activated) showed that in the control group, a baseline spontaneous activity of 6.05 spikes/s (1.27–11.7) increased significantly by 1.6 spikes/s (0–9.12) 1 h after CSD and by 1.98 spikes/s (0.1–6.02) 2 h after CSD (χ^2^ = 11.7, DF = 2, *P =* 0.0009, Friedman test). *Post hoc* (Tukey HSD) comparisons between baseline and 1 and 2 h post-CSD onset yielded *P-*values of 0.003 and 0.0001, respectively [Fig. 5C(i)]. In contrast, in the treatment group, spontaneous activity remained unchanged after CSD. The neurons’ baseline firing rate of 5.75 spikes/s (0.96–11.36) did not change significantly at 1 h [decrease of 1.07 spikes/s (−3.88 to 0) or 2 h (decrease of 0.37 spikes/s) (−3.87 to 4.63) after CSD (χ^2^ = 3.2, DF = 2, *P =* 0.18, Friedman test)] [Fig. 5C(ii)].

### Responses to mechanical stimulation of the dura before and after CSD

#### High threshold neurons

**Figure 6:**
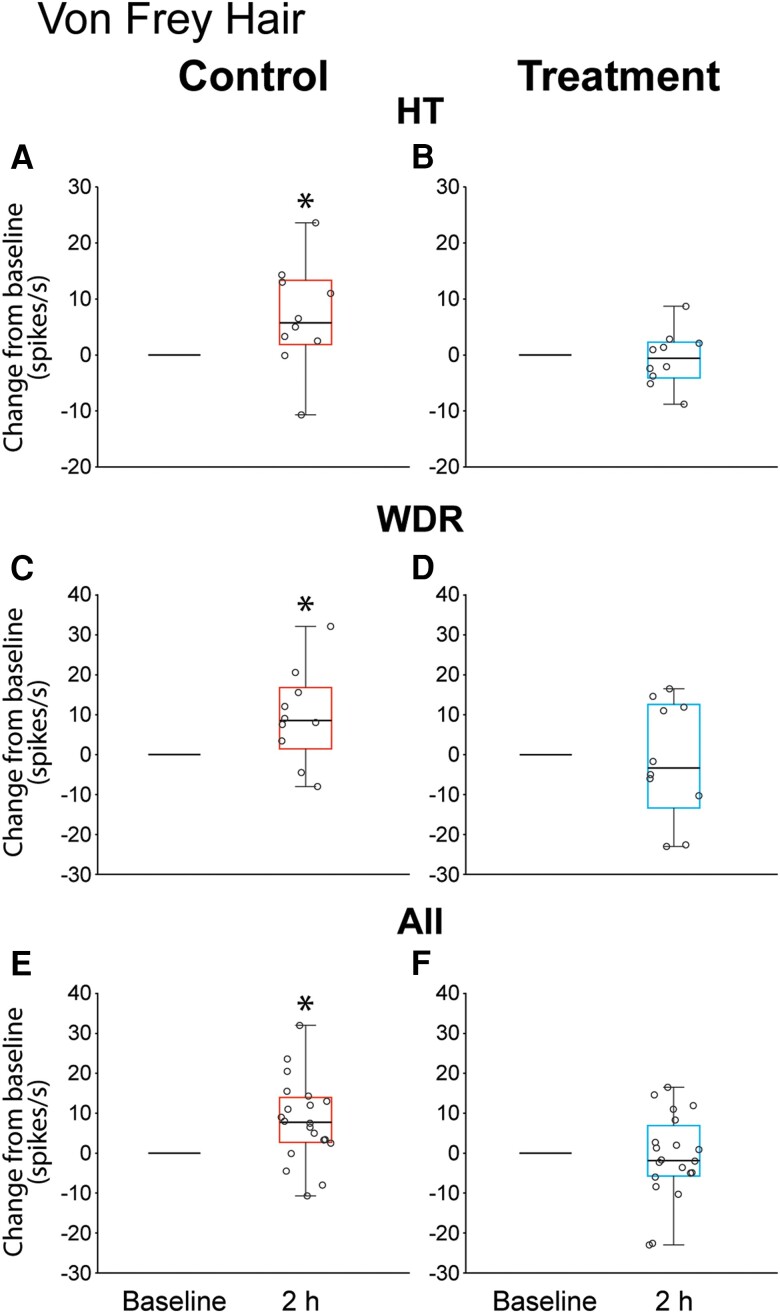
**Changes from baseline in response to dural indentation 2 h after CSD induction.** (**A**, **C** and **E**) Animals treated with saline. (**B**, **D** and **F**) Animals treated with atogepant. Box and whisker plots depict median and IQR (**A**–**D**, 10 neurons per group; **E** and **F**, 20 neurons per group). Scatter plots depict changes from baseline of individual neurons. *P < 0.05 Wilcoxon signed rank test. CSD = cortical spreading depression; HT = high threshold; WDR = wide dynamic range.

In the control group, baseline responses to dural indentation with calibrated VFH monofilament (4.1 g) [18.3 spikes/s (7.4–29.25)] increased significantly [by 5.75 spikes/s (1.85–13.32)] 2 h after CSD (*P =* 0.037, *Z* = 2.08, *n*_1_ = 10, *n*_2_ = 10, Wilcoxon signed rank test) (Fig. 6A). In the treatment group, baseline responses to dural indentation with the same VFH monofilament [18.65 spikes/s (7.05–23.45) decreased (rather than increased), though insignificantly, by 0.55 spikes/s (−3.92 to 2.17) 2 h after CSD (*P =*0.57, *Z* = 0.61, *n*_1_ = 10, *n*_2_ = 10, Wilcoxon signed rank test] (Fig. 6B). At the individual level, 8/10 (80%) neurons in the control group exhibited enhanced responses to dural stimulation after the CSD whereas in the treatment group only one neuron (10%) exhibited such enhanced responses.

#### Wide dynamic range neurons

In the control group, baseline responses to dural indentation with calibrated VFH monofilament (4.1 g) [9.0 spikes/s (6.12–14.07)] increased significantly [by 8.50 spikes/s (1.42–16.75)] 2 h after CSD (*P =* 0.029, *Z* = 2.14, *n*_1_ = 10, *n*_2_ = 10, Wilcoxon signed rank test) (Fig. 6C). In the treatment group, baseline responses to dural indentation with the same VFH monofilament [10.9 spikes/s (8.82–30.6)] remained unchanged [−3.35 spikes/s (−13.37 to 12.57) 2 h after CSD (*P =* 0.92, *Z* = 0.15, n_1_ = 10, n_2_ = 10, Wilcoxon signed rank test)] (Fig. 6D). At the individual level, 7/10 (70%) neurons in the control group exhibited enhanced responses to dural stimulation after the CSD whereas in the treatment group 4/10 (40%) exhibited such enhanced responses.

#### All neurons

In the control group, baseline responses to dural indentation with calibrated VFH monofilament (4.1 g) [9.55 spikes/s (7.2–25.5)] increased significantly [by 7.75 spikes/s (2.7–13.97)] 2 h after CSD (*P =* 0.002, *Z* = 2.89, *n*_1_ = 20, *n*_2_ = 20, Wilcoxon signed rank test) (Fig. 6E). In the treatment group, baseline responses to dural indentation with the same VFH monofilament [13.15 spikes/s (8.47–25.15)] remained unchanged [−1.85 spikes/s (−5.75 to 6.9) 2 h after CSD (*P =* 0.65, *Z* = 0.46, *n*_1_ = 20, *n*_2_ = 20, Wilcoxon signed rank test] (Fig. 6F).

### Responses to mechanical stimulation of the skin before and after CSD

#### High threshold neurons

**Figure 7:**
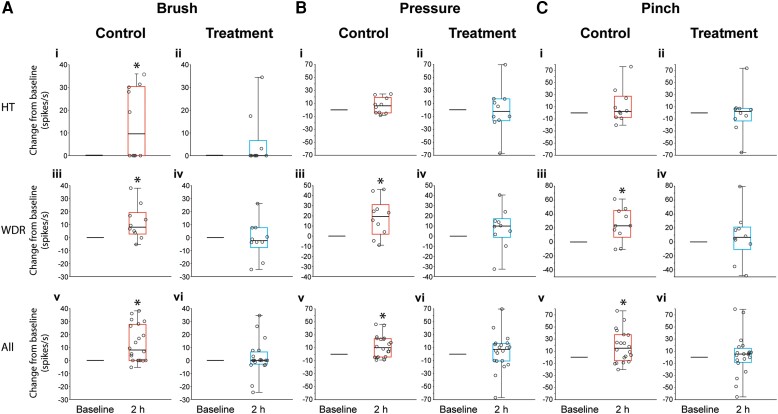
**Changes from baseline in responses to mechanical stimulation of the skin. (A)** Brush, (**B**) pressure and (**C**) pinch 2 h after cortical spreading depression (CSD) induction in animals treated with saline (control) and atogepant (treatment). Box and whisker plots depict median and IQR (**i**–**iv**, 10 neurons per group; **v** and **vi**, 20 neurons per group). Scatter plots depict changes from baseline of individual neurons. *P < 0.05 Wilcoxon signed rank test.

In the control group, baseline responses to skin stimulation with brush, pressure and pinch were [brush: 0 spikes/s (0–0); pressure: 19.6 spikes/s (5.92–30.47); pinch: 34.25 spikes/s (23.47–41.02)]. Two hours after CSD, responses to brush increased significantly [by 9.6 spikes/s (0–30.42), *P =* 0.0436, *Z* = 2.02, *n*_1_ = 10, *n*_2_ = 10], whereas responses to pressure [increased by 6.25 spikes/s (−4.66 to 19.22), *P =* 0.23, *Z* = 1.27, *n*_1_ = 10, *n*_2_ = 10] and pinch [increased by 2.4 spikes/s (−7.7 to 27.5), *P =* 0.37, *Z* = 0.96, *n*_1_ = 10, *n*_2_ = 10] remained unchanged (Fig. 7A(i), B(i) and C(i)]. In the treatment group, baseline responses to skin stimulation with brush, pressure and pinch were [brush: 0 spikes/s (0–0); pressure: 9.84 spikes/s (0–31.27); pinch: 24.85 spikes/s (10.92–40.71)]. Two hours after CSD, responses to brush [0 spikes/s (0–6.66), *P =* 0.25, *Z* = 1.60, *n*_1_ = 10, *n*_2_ = 10], pressure [−2.5 spikes/s (−16.81 to 16.77), *P =* 1.0, *Z* = 0.05, *n*_1_ = 10, *n*_2_ = 10] and pinch [2.50 spikes/s (−13.35 to 7.37), *P =* 1.0, *Z* = 0.05, *n*_1_ = 10, *n*_2_ = 10] remained unchanged [Fig. 7A(ii), B(ii) and C(ii)].

#### Wide dynamic range neurons

In the control group, baseline responses to skin stimulation with brush, pressure and pinch were [brush: 13.25 spikes/s (5.67–15.22); pressure: 15.55 spikes/s (5.52–22.07); pinch: 21.25 spikes/s (13.07–30.15)]. Two hours after CSD, responses to brush [increased by 8.05 spikes/s (2.72–19.30), *P =* 0.01, *Z* = 2.29, *n*_1_ = 10, *n*_2_ = 10], pressure [increased by 19.40 spikes/s (1.90–31.10), *P =* 0.01, *Z* = 2.29, *n*_1_ = 10, *n*_2_ = 10] and pinch [increased by 23.20 spikes/s (6.80–45.0), *P =* 0.009, *Z* = 2.49, *n*_1_ = 10, *n*_2_ = 10] increased significantly [Fig. 7A(iii), B(iii) and C(iii)]. In the treatment group, baseline responses to skin stimulation with brush, pressure and pinch were [brush: 26.75 spikes/s (14.85–35.95); pressure: 29.92 spikes/s (19.75–39.10); pinch: 38.35 spikes/s (26.20–53.43)]. Two hours after CSD, responses to brush [decreased by 2.35 spikes/s (−7.70 to 7.62), *P =* 0.76, *Z* = 0.35, *n*_1_ = 10, *n*_2_ = 10], pressure [increased by 9.95 spikes/s (−1.17 to 17.23), *P =* 0.16, *Z* = 1.47, *n*_1_ = 10, *n*_2_ = 10] and pinch [increased by 6.63 spikes/s (−10.95 to 21.12), *P =* 0.37, *Z* = 0.96, *n*_1_ = 10, *n*_2_ = 10] remained unchanged [Fig. 7A(iv), B(iv) and C(iv)].

## Discussion

**Figure 8:**
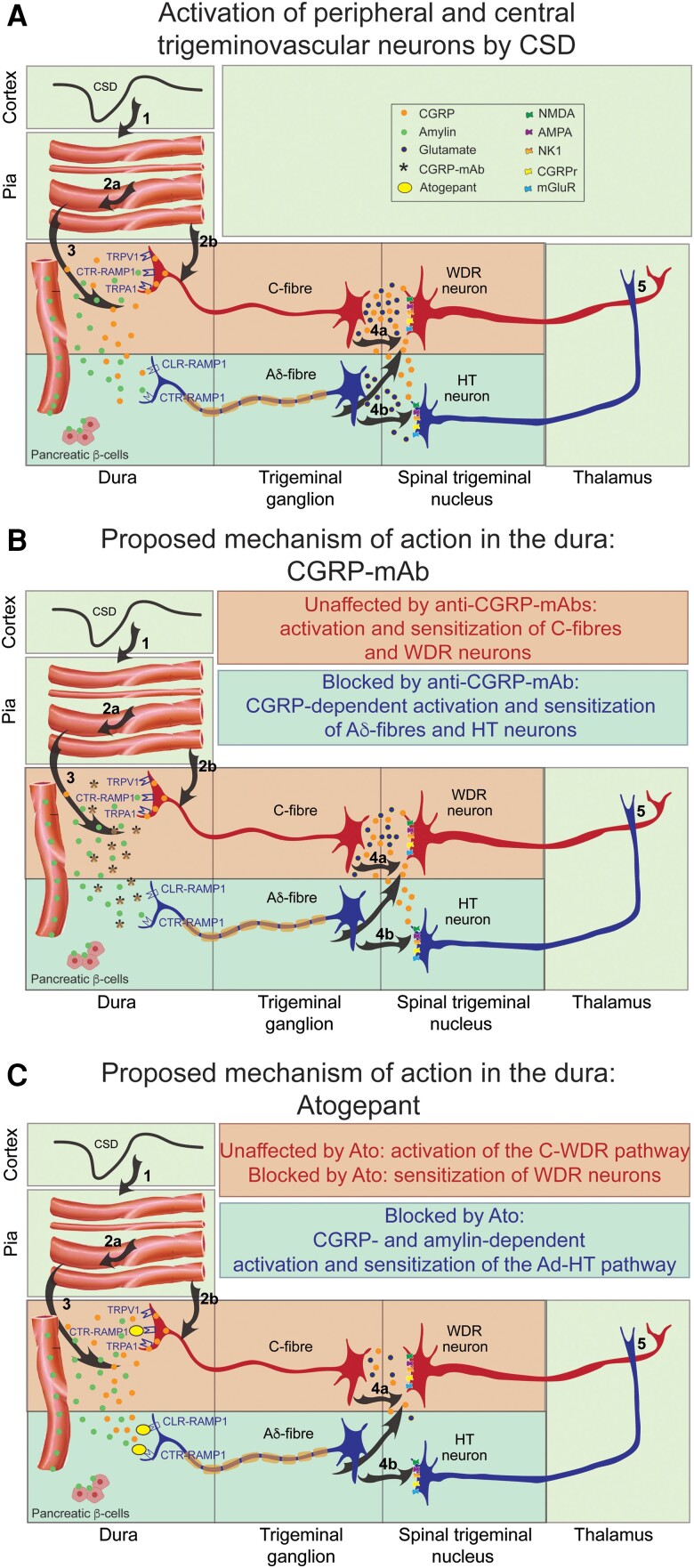
**Hypothesized differences between mechanisms of action of atogepant (a small molecule CGRP-receptor antagonist and Amy1-receptor antagonist) and fremanezumab (an anti-CGRP-mAb)**. (**A**) Proposed sequence of events leading to cortical spreading depression (CSD)-induced calcitonin gene-related peptide (CGRP)-independent early activation of C-fibres and wide dynamic range (WDR) neurons, and CGRP-dependent delayed activation of Aδ-fibres and high threshold (HT) neurons. (**B**) Proposed mechanism of action of CGRP-mAb (monoclonal antibody) in the dura. This proposal integrates results from our previous studies,^31,32^ showing that fremanezumab prevents the delayed activation and sensitization of Aδ-fibres and HT neurons but not the early activation and sensitization of C-fibres and WDR neurons. (**C**) Proposed mechanisms of action of Atogepant (Ato) in the dura. This proposal integrates results from the current and our previous study,^30^ showing that atogepant attenuates the early activation of C-fibres and delayed activation of the Aδ-fibres. The scientific framework presented in **A** is based on current evidence for (i) presence of CGRP in peptidergic C but not Aδ-fibres^36,42^; (ii) presence of CGRP receptors in Aδ but not C-fibres^34^ and in WDR and HT neurons^43,44^; (iii) CGRP release from central branches of C-fibres in the medullary and upper cervical dorsal horn^29,45^; (iv) a distinction between physiological and molecular processes that govern activation versus sensitization of central nociceptive neurons; (v) CGRP predominant role in neuronal sensitization^46,47^; (vi) predominant contribution of C-fibre nociceptors to the development of central sensitization^48^; (vii) presence of AMY 1 receptors in both Aδ and C-fibres^49,50^; (viii) amylin analogue ability to provoke migraine-like headache^51^; (ix) high level of amylin in the plasma of chronic migraine patients^52^; and (x) speculation about pancreatic (b-cells) origin of amylin in the dura.^53,54^ Based on the above, we are proposing that by neutralizing the CGRP peptide, anti-CGRP monoclonal antibodies can prevent activation of both the CLR-RAMP1(CGRP receptor) and CTR-RAMP1 (Amy1 receptor) by CGRP, but not the activation of the CTR-RAMP1 by amylin (illustrated in **B**). In contrast, atogepant’s ability to block the CLR-RAMP1 as well as the CTR-RAMP1^55-57^ can prevent their activation by CGRP as well as amylin (illustrated in **C**). In the context of migraine, this figure illustrates the potential importance of reducing C-fibre input and consequential CGRP release in nociceptive laminae of the spinal trigeminal nucleus as such interference can prevent the development and establishment of central sensitization—a physiological state tightly correlated with disease progression.^58,59^ (Please note that for simplicity, we did not mark presence of CGRP receptors in presynaptic terminals and consequently, did not discuss the possibility that activation of these receptors can contribute to the development of central sensitization by enhancing presynaptic glutamate release in the dorsal horn). Numbers in bold are explained at the bottom. **1.** CSD; **2a.** Vascular response; **2b.** activation of C-fibers. **3.** Activated C-fiber release of CGRP(and possibly adrenomedullin) in the dura, leading to activation of Aδ-fibers; **4a.** Activated C-fibers release glutamate and CGRP in the dorsal horn, leading to activation and sensitization of WDR neurons and sensitization of HT neurons; **4b.** Activated Aδ-fibers release glutamate that activates HT neurons; **5.** Activated WDR and HT neurons send signals to the thalamus. AMPA = ionotropic glutamate receptors; CLR-RAMP1 = CGRP receptor; CTR-RAMP1 = amylin (Amy1) receptor; mGluR1 = metabotropic glutamate receptor 1; NK1 = neurokinin-1 (substance P) receptor; NMDA = *N*-methyl-D-aspartate (glutamate) receptor.

Single cell analysis of atogepant pretreatment effects on CSD-induced activation and sensitization of central trigeminovascular neurons in the STN revealed the ability of this small molecule CGRP receptor antagonist to prevent activation of nearly all HT neurons and their enhanced responses to stimulation of the dura and facial skin. Atogepant's ability to prevent activation and sensitization of these neurons is attributed to its preferential inhibitory effects on thinly unmyelinated Aδ-fibres^30^—the class of meningeal nociceptors that express CGRP receptors and whose central branches converge mainly on HT neurons (Fig. 8C).^60,61^ In contrast, single cell analysis of atogepant pretreatment effects on CSD-induced activation and sensitization of WDR neurons revealed an overall inability to prevent their activation or sensitization to dural stimulation. Atogepant's inability to prevent activation of these neurons is attributed to its lesser inhibitory effects on the unmyelinated C fibres^30^—the class of meningeal nociceptors whose central branches converge mainly on WDR neurons (Fig. 8C).^61^ Unexpectedly however, in spite of atogepant's inability to prevent activation of WDR neurons, it prevented their sensitization to facial stimulation. As enhanced responses to dural stimulation can reflect sensitization of peripheral and/or central trigeminovascular neurons whereas enhanced responses to stimulation of the facial skin reflects sensitization of central but not peripheral neurons,^62-64^ we concluded that atogepant was able to prevent central sensitization in this class of neurons. A possible explanation for atogepant's ability to prevent central sensitization in the WDR neurons is found in the group analysis. This analysis revealed that while atogepant pretreatment did not prevent activation of individual WDR neurons, it significantly attenuated the overall increase in their firing after occurrence of CSD and equally important, their enhanced responses to dural stimulation. These findings are attributed to atogepant's partial inhibitory impact on the responsiveness of the unmyelinated C-fibre meningeal nociceptors (Fig. 8).^30^ Molecular and physiological processes that govern neuronal activation versus sensitization can explain how reduction in CGRP-mediated slow but not glutamate-mediated fast synaptic transmission between central branches of meningeal nociceptors and nociceptive neurons in the STN can prevent their sensitization but not activation.^65-67^

Recently, we showed that combined onabotulinumtoxinA/atogepant pretreatment prevents CSD-induced activation and sensitization of nociceptive HT and WDR trigeminovascular neurons in the spinal trigeminal nucleus.^40^ One of the more puzzling findings of that study was that this combination therapy had a more pronounced inhibitory effect on the WDR (where it prevented activation of all neurons) than the HT neurons. Based on published data that were available then, we suggested that the reduction in CSD-induced activation of the HT neurons was secondary to the preferential inhibitory effects of drugs that inhibit CGRP signalling on thinly-myelinated Aδ fibres in the dura,^31,32^ whereas the blockade of CSD-induced activation of the WDR neurons was secondary to the preferential inhibitory effects of onabotulinumtoxinA on the unmyelinated C fibres in the dura.^68-70^ But given that CGRP inhibitors act systemically and can theoretically inhibit all dural Aδ fibres, whereas onabotulinumtoxinA is administered and acts locally and thus is unlikely to inhibit all dural C fibres, we could not explain the more pronounced inhibitory effect of this dual therapy on the WDR neurons. However, a year later, we showed that unlike the selective inhibition of CSD-induced activation and sensitization of Aδ fibres by fremanezumab, atogepant produced immediate but brief inhibition of firing in the unmyelinated C fibres as well as delayed and prolonged inhibition of neuronal firing in the Aδ fibres. Based on the presence of CGRP receptors in Aδ but not C fibres,^34,36,71^ we then proposed that atogepant's unexpected inhibitory effects on the C fibres may be mediated by its ability to bind to the AMY1 receptor (presence of *AMY1* receptors in the trigeminal ganglion is confirmed by immunohistochemical labelling of CTR^49^ and RT-PCR detection of *RAMP1*^50^) on this class of meningeal nociceptors^49,55,56,72-74^ and partially block their activation by CGRP^73^ or amylin^75^ (Fig. 8A and C). Conceptual evidence for amylin's role in migraine includes reports of an amylin analogue's (pramlintide) ability to trigger a migraine-like headache in patients,^51^ and its high plasma level in chronic migraine patients.^52^ Given that the origin of amylin in the plasma is pancreatic β-cells,^53^ and that their ability to self-regulate amylin secretion is commonly determined/affected by consumption of glucose, fats and proteins,^53^ stress^54^ and melatonin (a hormone primarily known for its role in regulating sleep-wake cycles) secretion,^76^ it is tempting to hypothesize that amylin secretion may play a role in the mechanisms by which migraine is triggered by food, stress and sleep.

Two sets of findings in the current study enable us to understand better the complexity of atogepant's mechanism of action in migraine prevention. The first is atogepant's ability to prevent activation of HT but not WDR neurons by CSD, and the second is atogepant's ability to prevent the development of central sensitization in the same WDR neurons in which it could not prevent activation. As the rate of activation of HT neurons in the current study was identical to their rate of activation in the combination study (activation rate in both studies was 80% in the control group and 20% in the treatment group), it is likely that the inhibition of the HT neurons was mediated exclusively by the atogepant. In contrast, as the rate of activation of the WDR neurons in the current study was far inferior to their rate of activation in the combination study (current study: 70% in the control group versus 50% in the treatment group; combination study: 70% in the control group versus 0% in the treatment group), it is reasonable to conclude that the prevention of activation of this class of neurons was mediated almost exclusively by the onabotulinumtoxinA.

Current understanding of physiological processes that regulate activation versus sensitization of central nociceptive neurons in the dorsal horn can explain atogepants’ ability to prevent sensitization but not activation of WDR neurons. In principle, activation that forms the foundation of pain intensity, location and duration is thought to be the result of normal nociceptive transduction, conduction and fast synaptic transmission between central branches of nociceptors and nociceptive (trigeminovascular) neurons in the medullary dorsal horn. The fast synaptic transmission required to activate the dorsal horn neurons is mediated mainly by glutamate (released from unmyelinated C fibres) (Fig. 8A–C)—induced fast excitatory postsynaptic potentials through its action on AMPA receptor.^77,78^ In contrast, sensitization of central nociceptive neurons—the process by which prolonged and intense C-fibre input modifies their excitability, sensitivity and responsivity by eliciting post-translational changes in membrane-bound receptors^48^—depends mainly on prolonged period of high stimulus intensity and slow synaptic transmission. In general, such transmission can involve the release of molecules such as CGRP, substance P or glutamate from unmyelinated C fibres that in turn sensitize the central neurons by acting on their CGRP,^46,47^ NK1^79,80^ NMDA^81,82^ and group 1 mGluR^83-85^ receptors (Fig. 8, TG-STN interactions; receptors are shown as postsynaptic, but could also be presynaptic). Presence of these receptors in both laminae I and V of the spinal trigeminal nucleus^43,44,86-93^ provides the anatomical substrate for this proposal. In the context of the current study, we propose that atogepant’ ability to attenuate the duration and overall intensity of the nociceptive inputs from the meningeal C fibres to the spinal trigeminal nucleus (Fig. 8C) is key to its ability to prevent the formation of central sensitization. Given central sensitization's role in migraine progression,^58,59^ resistance to successful termination of acute migraine attacks with triptans^94,95^ and prevention with CGRP mAbs,^96^ atogepant's impact on central sensitization is emphasized. This conclusion further accentuates the mechanistic differences between atogepant (Fig. 8C) and mAbs that neutralize the CGRP molecule (Fig. 8B).

Theoretically, sensitization of central neurons to dural stimulation can have a peripheral as well as central origin, and the blockade of this sensitization by atogepant in the HT neurons could, in principle, result in part from blockade of sensitizing action of CGRP on the dural Aδ fibres. Support to such a possibility may be found in studies showing that CGRP can affect intracellular signalling cascades in trigeminal ganglion cells^97^ and produce subthreshold effects on membrane currents in *in vitro* recordings from dorsal root ganglion cells.^98,99^ Our interpretations of the findings, however, do not give much weight to this possibility because *in vivo* studies to date have failed to find evidence of sensitizing effects (increased firing) of CGRP on nociceptors^100^ or hyperalgesic effects in humans.^101^

One of the more intriguing findings of the current study was that atogepant was able to prevent the sensitization of WDR neurons and attenuate their response magnitude. It is intriguing because it is the first documented mechanistic difference between the effects of fremanezumab on central trigeminovascular (in this case, WDR) neurons responses to CSD and the effects of atogepant. The translatability of these differences to clinical practice, however, is unclear as currently available published data on comparable effectiveness are lacking.^102^ In principle, a similar argument must be applied to data showing functional evidence for small molecule CGRP receptor antagonists (gepants) but not anti-CGRP mAbs’ ability to prevent nociceptive behaviour in female mice by acting centrally.^103^ Regarding this study, it must be noted that in the current study, atogepant (unlike fremanezumab), prevented the sensitization of the WDR neurons but not their activation—suggesting that the possible central effect of the gepants is also insufficient to prevent the initiation of the headache phase of migraine (which is remarkably different from sensitization). In the absence of robust clinical trial and real-world data that compare all products within the class and show superiority of gepants, the significance of these animal studies remains unclear. If, however, future head-to-head studies could reveal improved efficacy (in either primary or secondary end points), the translatability of the current findings will need to be re-evaluated.

### Caveats

A limitation of this study is that it was carried out in males only. We have found that it is more difficult to maintain stable physiological parameters and obtain well isolated neuronal recordings in females—factors critical for establishing reliability in the present experimental paradigm. In addition, our previous study on atogepant effects on the meningeal nociceptors was also carried out in males (which makes comparisons between the two studies more relevant). Our previous studies with both sexes have found no basic differences in CSD responses and drug effects,^31,32^ but nonetheless it will be useful in future studies to confirm the present results in females.

## Data Availability

The data that support the findings of this study are available from the corresponding author, upon reasonable request.
